# Editorial: Innovative ways of targeting the RTK/RAS/RAF pathway

**DOI:** 10.3389/fonc.2026.1922564

**Published:** 2026-08-12

**Authors:** Alessandro Passardi, Maxim Noeparast, Zoran Todorovic

**Affiliations:** 1 Department of Medical Oncology, Scientific Institute of Romagna for the Study and Treatment of Tumors (IRCCS), Meldola, Italy; 2 Department of Hematology and Clinical Oncology, University Medical Center and Medical Faculty, Augsburg University, Augsburg, Germany; 3 Department of Pharmacology, Clinical Pharmacology and Toxicology, and University Clinical Medical Center "Bezanijska kosa", Faculty of Medicine, University of Belgrade, Belgrade, Serbia

**Keywords:** MAPK pathway, oncogenic signaling, RTK/RAS/RAF axis, targeted therapy, therapeutic resistance

The receptor tyrosine kinase (RTK)/RAS/RAF/MEK/ERK (mitogen-activated protein kinase, MAPK) signaling cascade is a pivotal axis in normal and cancer cell biology, playing a critical role in tumour initiation, progression, and treatment resistance (1, 2). Despite substantial progress in targeting canonical alterations, most notably *EGFR*-activating mutations and *BRAF V600E* mutations, durable clinical control in RTK/RAS/RAF-driven malignancies remains an unmet need (3).

In recent years, the therapeutic landscape has expanded beyond classical actionable alterations to encompass a broader range of genomic events, including KRAS G12C, atypical EGFR, and non-V600E BRAF mutations, with emerging evidence suggesting a potential role for CRAF (RAF1) in human cancer (3–6).

The clinical development of KRAS G12C inhibitors has been a major step forward in targeting RAS-driven tumours; however, clinical experience has also shown that responses are often temporary and that resistance can emerge quickly through adaptive mechanisms (7–9). At the same time, other KRAS mutations beyond G12C, atypical EGFR and BRAF variants, and alterations in CRAF remain largely unexplored therapeutic opportunities (4, 10, 11). KRAS G12D inhibitors are also currently under clinical investigation (12).

In addition to therapies designed to inhibit oncogenic signaling, combinations of MAPK-targeted agents with immunotherapy represent another emerging strategy (13).

Interestingly, in recent years a new and somewhat counterintuitive aspect of the RTK/RAS/RAF pathway has been explored in preclinical models. While most efforts have historically focused on inhibiting this key oncogenic pathway, there is increasing evidence that cancer cells may also be vulnerable to its overactivation (2, 14). This paradoxical phenomenon could represent an underappreciated aspect of tumour biology. However, this concept is still in its early stages and requires further validation before any clinical application.

The MAPK pathway is a highly interconnected signaling network in which dynamic feedback loops, pathway redundancy, and crosstalk with upstream and downstream effectors contribute to therapeutic resistance. Increasing evidence indicates that resistance to targeted therapies is frequently driven by reactivation of MAPK signaling or activation of compensatory parallel pathways (3). This has prompted the exploration of novel therapeutic strategies, including vertical pathway inhibition, combination regimens targeting multiple nodes of the RTK/RAS/RAF axis, and unconventional approaches such as modulation of scaffolding proteins and paradoxical MAPK activation (3, 15).

These findings underscore the urgent need for next-generation inhibitors and rational combination strategies. This *R*esearch *T*opic was designed to capture the most recent advances in the field, with particular emphasis on atypical yet recurrent alterations in *EGFR*, *BRAF*, *KRAS*, and *RAF1*, as well as emerging translational and therapeutic strategies to expand the clinical actionability of the RTK/RAS/RAF pathway. The contributions included herein reflect both the biological complexity and clinical heterogeneity of this signaling network.

A clinically relevant example of the evolving therapeutic landscape is provided by the report of an exceptional response to furmonertinib in lung adenocarcinoma harbouring a HER2 exon 20 insertion mutation, highlighting the functional interplay between ERBB family alterations and downstream MAPK signaling dependency (16). This case emphasises how atypical receptor alterations may converge on RTK-dependent signaling vulnerabilities that are potentially targetable.

Complementing this observation, Mao et al. report a case of pulmonary enteric adenocarcinoma harbouring concurrent KRAS, TP53, and APC mutations with contralateral lung metastasis, illustrating the complexity of metastatic dissemination in KRAS-driven tumours and the cooperative role of co-occurring genomic alterations (17). This case underscores the importance of considering broader genomic context when interpreting KRAS-mutant disease biology.

Therapeutic targeting of the MAPK pathway is also shown by the combination of dabrafenib, trametinib, and pembrolizumab in a patient with BRAF V600E-mutant lung adenocarcinoma, suggesting the feasibility and potential benefit of combining vertical pathway inhibition with immune checkpoint blockade (18). This report supports the concept that deep suppression of MAPK signaling, when integrated with immunotherapy, may enhance clinical durability.

Beyond clinical cases, experimental modelling systems are also advancing the field. Costabile et al. developed an early-stage 3D fibroblast-featured tumour model that recapitulates key features of the tumour microenvironment, including gene expression programs associated with tumour progression and therapeutic resistance An early-stage 3D fibroblast-featured tumor model mimics the gene expression of the naïve tumor microenvironment, including genes involved in cancer progression and drug resistance (19). Such platforms provide valuable tools for investigating adaptive resistance mechanisms and evaluating combination therapies under physiologically relevant conditions.

At the interface of oncogenic signaling and metabolism, Defazio et al. demonstrated that *KRAS* mutations can drive transcriptional reprogramming of the peroxisome proliferator-activated receptor pathway in pancreatic adenocarcinoma (20). These findings expand the functional consequences of KRAS signaling beyond canonical MAPK activation and suggest additional metabolic vulnerabilities that may be therapeutically relevant.

Although not directly focused on RTK/RAS/RAF alterations, the report by Li et al. describing rare pancreatic involvement in Erdheim–Chester disease provides further evidence of MAPK pathway dysregulation in histiocytic neoplasms, reinforcing the broader relevance of this signaling cascade across oncologic and hematologic diseases (21).

Collectively, these contributions highlight several converging themes. First, RTK/RAS/RAF pathway alterations include a highly heterogeneous spectrum extending beyond canonical driver mutations. Second, effective therapeutic targeting requires integrated strategies that address pathway redundancy, feedback activation, and adaptive resistance. Third, tumour microenvironmental context and metabolic rewiring are emerging as key determinants of therapeutic response.

In conclusion, this *R*esearch *T*opic underscores a conceptual shift in targeting the RTK/RAS/RAF pathway: from single-node inhibition to integrated, multi-level therapeutic strategies that combine precise molecular stratification, vertical and horizontal pathway targeting, and modulation of tumour–microenvironment interactions. Continued integration of translational models and clinical observations will be essential to advance durable and broadly effective therapies for RTK/RAS/RAF-driven cancers.
